# Postoperative pain management in obstetrics and gynecology

**DOI:** 10.4274/jtgga.galenos.2020.2020.0024

**Published:** 2020-12-04

**Authors:** Henning Ohnesorge, Veronika Günther, Matthias Grünewald, Nicolai Maass, İbrahim Alkatout

**Affiliations:** 1 Clinic of Anesthesiology and Operative Intensive Medicine, University Medical Center Schleswig-Holstein, Kiel, Germany; 2 Clinic of Obstetrics and Gynecology, University Medical Center Schleswig-Holstein, Kiel, Germany

**Keywords:** Pain management, obstetrics, gynecology, nerve block, anesthesia

## Abstract

The efficiency and quality of postoperative pain management may be considered unsatisfactory in Europe, as well as in the United States. Notwithstanding our better understanding of the physiology of pain and the development of new analgesia procedures, the improvement in satisfaction of patients has not be enhanced to the same degree. Obstetrics and gynecology are no exception to this statement. In fact, obstetrics and gynecology are surgical departments in which patients experience the greatest severity of postoperative pain. Current concepts of postoperative pain management are largely based on the administration of systemic non-opioid and opioid analgesics, supplemented with regional analgesia procedures and/or peripheral nerve blockades and, in some cases, the administration of other pain-relieving pharmaceutical agents. Based on the existing body of evidence, it would be appropriate to develop procedure-related concepts of analgesia. The concepts are based on the special circumstances of the respective department, and the scheme of analgesia is aligned to the respective interventions. Generally, however, a surgeon’s individual experience in dealing with the procedures and substances could be more significant than the theoretical advantages demonstrated in preceding investigations.

## Introduction

Despite significant progress in our comprehension of the mechanisms of pain, pain physiology, and the pharmacology of effective analgesic substances, postoperative pain management remains a major challenge in medicine. Insufficiently treated postoperative pain impairs postoperative convalescence in many ways and thus also influences perioperative morbidity and the duration of hospital stays. Despite the advancements made in some areas with regard to the treatment of postoperative pain, the quality of postoperative pain management is generally unsatisfactory in Europe, as well as in the United States (1,2,3). This does not necessarily mean that major operations are associated with very intense pain and minor procedures involve less pain. Rather, it has been noted in patient surveys that analgesia is rather poor after routine and frequently performed operations, whereas - contrary to general expectations - major operations are rated positively by patients (4). One of the reasons for this discrepancy could be the patients’ expectations. Those who undergo major operations tend to anticipate and accept pain.

Postoperative pain management needs to be improved to a large extent in obstetrics and gynecology as well. This is reflected by the fact that the pain scores of patients in Departments of Obstetrics and Gynecology, German Hospitals are higher than those at all other surgical departments (5). Gynecological operations are also reported to cause high levels of pain. Especially open operations in the uterus are associated with severe pain; the latter is comparable with pain scores after spondylodesis. Thus, open operations in the uterus are followed by the highest pain scores in Germany. However, complex operations in the female breast and supposedly minor laparoscopic interventions such as adenectomies are also associated with significant postoperative pain (6). In fact, pain scores in the afore-mentioned settings exceed those reported after major operations in the upper abdomen (such as partial resection of the liver) (5).

Current concepts of postoperative pain therapy are usually based on a combination of various analgesics and/or regional anesthesia to achieve a balanced analgesia regimen and adequate pain relief while causing acceptable side effects.

## Aims of postoperative pain management

The foremost aim of pain management is not to alleviate the intensity of pain but to reduce the patient’s suffering. The principle that applies here is: “Suffering may be associated with the significance of pain to the same extent as it is with the intensity of pain. A persistent feeling of helplessness and hopelessness may be the basic cause of a patient’s suffering when he/she experiences chronic pain. This is reflected in high pain scores” (7). Extrapolated to the postoperative situation, this means that the patient’s satisfaction and well-being should be given greater attention (8). In an investigations of patients who had undergone a caesarean section alone, questions focusing on the patient’s well-being rather than pain were able to influence their feedback concerning postoperative pain and their desire to receive more analgesics (9).

Factors other than the severity of pain were also found to influence a patient’s postoperative well-being. Especially the occurrence of nausea and vomiting are significant factors. The possibility of independent mobilization, particularly going to the toilet and personal hygiene, having sufficient sleep at night, being able to dispense with drainages, catheters and intravenous accesses, and a largely normal oral intake of food are important in this context. In summary, in the postoperative phase patients wish to achieve sufficient control over their physical symptoms as well as restore their autonomy rapidly. Interestingly, the wishes of patients are very similar to the postoperative goals of the Enhanced Recovery after Surgery (ERAS) Programme (10).

Despite these long-term goals, the severity of postoperative pain must be recorded regularly. This is usually achieved with the aid of a numeric rating scale (NRS) extending from 0 (no pain) to 10 (worst pain). However, the use of the NRS requires a certain capacity for abstraction on the part of the patient. Basically, postoperative pain should be recorded regularly and after any type of pain therapy in order to evaluate the success of the respective measure.

## Systemic analgesics

### Non-opioid analgesics

**Figure 1:**
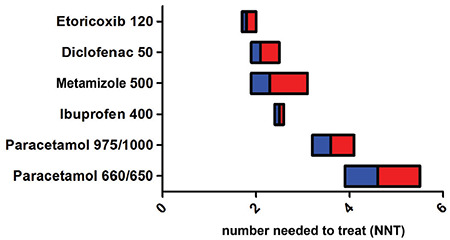
Analgesic potency of various non-opioid analgesics: Number needed to treat in order to reduce pain by at least 50% in patients with moderate to severe pain (red/blue: 95% confidence interval, median, based on (12)

Non-opioid analgesics constitute the basis of analgesia in postoperative pain management. The regular administration of individual non-opioids, such as paracetamol, traditional non-steroidal anti-inflammatory drugs (NSAIDs), selective cyclooxygenase-2 (COX-2) inhibitors or metamizole in standard doses provides sufficient pain relief after interventions associated with mild or moderate pain (11). The analgesic potency of non-opioid analgesics after oral administration has been extensively investigated (12) (Figure 1). We lack similar investigations for intravenous forms of application. In patients undergoing operations that involve severe pain, the regular administration of non-opioid analgesics as part of a balanced concept of analgesia may contribute to a reduction of opioid doses as well as side effects, and improve the quality of analgesia.

### Paracetamol

Of all non-opioid analgesics, paracetamol is regarded as the substance with the least analgesic potency (Figure 1). The analgesic efficacy of intravenous administration is more pronounced than that of oral or rectal administration. Paracetamol is well tolerated in therapeutic doses and has no relevant cardiovascular, gastrointestinal, or renal side effects. It also has no clinically significant impact on the function of thrombocytes. However, the use of paracetamol is controversially discussed because of its limited therapeutic spectrum and the risk of irreversible liver damage in case of overdosage. The highest daily dose for oral or rectal administration is 100 mg/kg body weight, and for intravenous administration 4 g/24 h (for those with a body weight less than 50 kg the dose is 60 mg/kg BW). Previous damage to the liver, a glutathione deficiency, such as that caused by excessive alcohol consumption, or the induction of the CYP-450 enzyme system, are listed as contraindications for the use of paracetamol.

### Metamizole

In addition to its favorable pain-relieving effect, metamizole has spasmolytic effects which may intensify its analgesic effect, especially in cases of colic or convulsive pain. However, in view of the fact that metamizole could trigger agranulocytosis, its use in Germany is restricted to five indications:

1. Acute and severe pain after injuries and/or operations,

2. Colic,

3. Tumor pain,

4. Any other acute or chronic severe pain, provided other therapeutic measures are not indicated,

5. High fever that does not respond to other measures.

These indications do not necessarily include the postoperative use of metamizole after operations. Rather, they refer to its use in the event of anticipated or existing severe postoperative pain. Owing to the risk of shock reactions, the parenteral administration of metamizole is explicitly indicated only when oral administration is not feasible (13).

Although the incidence of metamizole-induced agranulocytosis is considered to be rather low compared to that of other pharmacological agents, explaining the risks of using metamizole to the patent prior to its intended use has become an avidly discussed issue again. Agranulocytosis may also occur after prolonged treatment with metamizole and after a period of several days following its last administration. Therefore, it would be meaningful to inform the patient after the administration of metamizole about potential early symptoms of metamizole-induced agranulocytosis (fever, throat pain, inflammatory changes in the mucous membranes).

Notwithstanding these limitations, metamizole remains an essential component of the concept of balanced postoperative pain management because of its high tolerability and low or non-existent organ toxicity. Therefore, metamizole still is the preferred non-opioid analgesic for postoperative pain management in German-speaking countries (14).

## Non-steroidal antiphlogistic drugs

Traditional non-selective COX inhibitors as well as selective COX-2 inhibitors are marked by their significant analgesic effect in postoperative pain management. However, the use of traditional NSAIDs and coxibs is limited because of their spectrum of side effects. These especially include cardiovascular, renal, and gastrointestinal effects. Basically, the risk of gastrointestinal events is markedly higher during the long-term intake of nearly all NSAIDs and coxibs (15). The latter rule out the use of NSAIDs or coxibs in nearly all patients with relevant cardiovascular risk factors (CHD, heart failure NYHA II-IV, peripheral arterial occlusive disease, cerebrovascular disease). The same considerations apply to the risk of gastrointestinal bleeding. Substances with a gastrointestinal risk profile are associated with a higher risk of cardiovascular events as well.

As these substances have an effect on renal function, their postoperative use is contraindicated, especially in patients with a pre-existing limitation of renal function or hypovolemia. The latter can never be ruled out after major operations.

Furthermore, when using non-specific NSAIDs, one must take into account the fact that the inhibition of COX-1 may lead to a disorder of thrombocyte function. Thus, these substances may be associated with a higher risk of hemorrhage after surgery. In a recent meta-analysis, however, no elevated incidence of hematoma or post-surgical hemorrhage was registered after plastic operations (including those in the breast) and the intake of NSAIDs (16).

## Opioid analgesics

In patients with severe postoperative pain that cannot be adequately controlled with non-opioid analgesics, opioids still are the gold standard in postoperative pain management. In Germany piritramide has been established as a standard medication, although we lack robust evidence of the superiority of this substance over other opioids such as morphine, fentanyl or sufentanil. Intravenous administration is advantageous in the short term because of its rapid efficacy and the easy titration of these substances.

Opioids are marked by their favorable analgesic effect and the absence of organ toxicity. However, the spectrum of acute side effects of opioids in the postoperative phase is of considerable relevance. The administration and dosage of opioids for postoperative pain management is one of the major risk factors for the occurrence of nausea and vomiting. Furthermore, the inhibition of bowel motility, which occurs frequently under opioid treatment, is responsible for the delayed postoperative restoration of normal gastrointestinal function. The deleterious effects of opioids on respiratory depression in case of overdosage are a matter of great concern (17). The highly variable need for opioids in patients and the absence of predictive tools to address this problem are further difficulties (4). Thus, the administration of opioids in the postoperative phase is usually titrated according to the individual patient’s needs.

**Figure 2:**
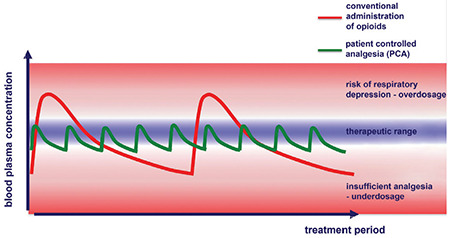
Theoretical course of plasma levels after conventional administration of opioids by nursing staff (infrequent application, higher doses) compared to patient-controlled application (PCA, small dose, frequent administration)

Opioids are titrated by the nursing staff and the substance is usually given in the form of short intravenous infusions, but these may be associated with the risk of relative overdosage. Therefore, if a patient needs opioids regularly, it would be advisable to use patient-controlled application systems patient-controlled analgesia (PCA). As the latter is administered frequently in smaller individual doses, this form of analgesia is associated with a lower risk of overdosage (Figure 2). Simultaneously, the use of PCA systems improves the quality of postoperative pain management (18).

The disadvantage of traditional PCA systems that permit intravenous administration of an opioid through a pump system is the indispensable need for an intravenous access and the consequent limitation of the patient’s mobility. A new system that permits patient-controlled administration of sufentanil sublingual microtablets is an alternative that, according to preliminary data, provides at least equivalent pain relief as intravenous PCA; it causes no limitation of the patient’s mobility and provides a greater degree of satisfaction for patients (19).

**Figure 3:**
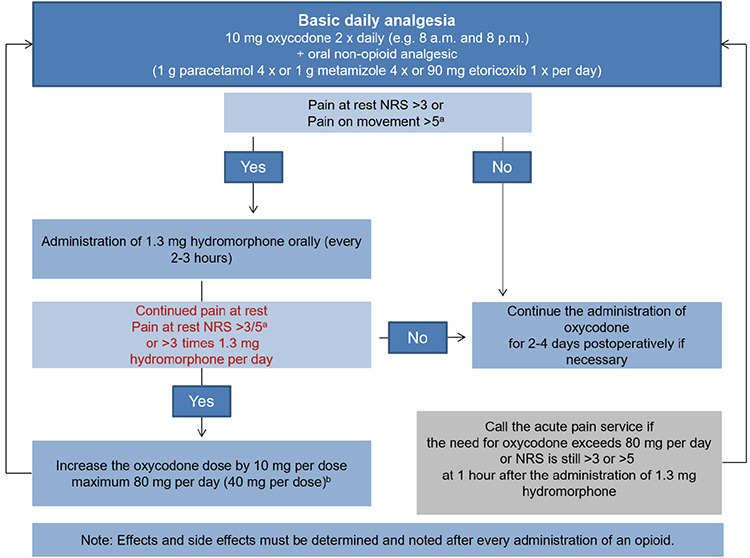
Concept of postoperative pain management: basic treatment with non-opioid analgesics and retarded opioids, and need-oriented treatment with non-retarded oral opioids [extracted from (47)], “numeric rating scale”. ^a^The reason for existing or increasing postoperative pain should be investigated by the surgeon in charge of the patient’s treatment.^b^The success or failure of any change in basic analgesia must be determined. NRS: Numeric rating scale

The perioperative use of retarded opioids is a further alternative to the purely need-oriented administration of opioids. The notion of administering a fixed dose of a retarded opioid does contradict the observation that the postoperative requirement of opioids varies very markedly from one patient to another. Therefore, recent American guidelines for postoperative pain therapy explicitly advise against the use of long-acting opioids in the early postoperative phase (20). The summaries of product characteristics for retarded opioids also clearly mentions that the substances should not be administered preoperatively or during the first 12-24 hours post-surgery. Nevertheless, clinical experience concerning the long-term administration of retarded opioids and the need-oriented administration of a non-retarded oral opioid has been quite favorable (21) (Figure 3).

## Gabapentinoids

In addition to traditional non-opioid and opioid analgesics, particularly gabapentin and pregabalin have been established in the management of postoperative pain. The preoperative administration of these substances, which are approved for the treatment of chronic neuropathic pain, appears to reduce the need for opioids as well as the incidence of nausea and vomiting after breast surgery and open hysterectomy (22,23). The effective dose remains unclear. The use of higher doses is quite evidently associated with a sedative effect.

## Systemic local anesthetics

The perioperative systemic administration of lidocaine is, in part, propagated as the “poor man’s epidural” because initial studies have shown a marked effect on the postoperative need for analgesics, the duration of gastrointestinal atony, and the incidence of opioid-related undesirable effects. In a recent Cochrane analysis (24), the effect of this procedure could not be clearly proven in comparison with placebo or traditional epidural analgesia. Therefore, the results of further studies are required before this procedure can be recommended as a standard for the management of perioperative pain.

## Regional anesthesia procedures

For many operations in obstetrics and gynecology, regional anesthesia procedures can be used to reduce the patient’s need for systemic analgesics and simultaneously improve the management of postoperative pain. The spectrum extends from wound infiltrations and peripheral nerve blockades to analgesia procedures in the vicinity of bone marrow, especially epidural and peridural analgesia.

## Wound infiltration

Wound infiltrations can be performed as a single-shot procedure or a continuous procedure, usually through a subcutaneous catheter connected to an elastomeric pump. While wound infiltrations in patients undergoing general and traumatological surgery have shown marked effects, the data reported in the obstetric and gynecological setting have been disappointing. Wound infiltrations as a means of postoperative pain management are recommended neither in laparoscopic surgery of the lower abdomen nor in surgery on the female breast (25,26). After caesarean section, wound infiltrations may reduce the need for opioids without influencing the incidence of opioid-related undesirable effects (27).

## Peripheral nerve blockade

Various types of peripheral nerve blockades may be used for postoperative pain management in obstetrics and gynecology. Paravertebral blockades (PVB) and pectoral nerve blockades (PECS I + II) in breast surgery, as well as the transversus abdominis plane (TAP) block in surgery of the lower abdomen are worthy of mention.

## Paravertebral blockade

PVB are regarded as a unilateral alternative to epidural analgesia procedures and are mainly used in the chest. After injection of local anesthetics in the paravertebral space, the anesthetist administers analgesia that usually reaches several segments and thus influences the roots of spinal nerves. PVB’s have been successfully used for many years in breast surgery as a means of perioperative pain management (28). It should be noted that a PVB may also be used to reduce the occurrence of chronic pain after breast surgery in women (29). The risk of undesirable effects (nerve injury, pneumothorax, vessel puncture) is considered negligible (28). However, a disadvantage is that PVB must be administered preoperatively or postoperatively with the patient in sitting position and wide awake. This reduces the acceptance of the procedure.

## Pectoral nerve block

The pectoral fascia block was first described in 2011 as an alternative to PVB (30). This was followed by various modifications of the procedure. Currently, a combination of fascia blocks between the pectoralis major and pectoralis minor muscles (PECS I block), and the pectoralis minor and serratus anterior muscles (PECS II block) at the level of the fourth rib has been established as a standard procedure. This combination appears to be as effective as a PVB (31). Its advantages are that it has a low risk profile even when administered after the induction of general anesthesia in a supine position (32).

## Transversus abdominis plane block

TAP block is a field block, similar to PECS. It encompasses the abdominal wall branches of the thoracic spine and lumbar spine nerve roots, and is used as a means of perioperative analgesia, especially when performing surgery in the lower abdomen. A depot of a local anesthetic is injected into the layer of fascia between the internal oblique muscle and the transversus abdominis muscle, in the medioclavicular line between the costal arch and the iliac crest. In gynecology, operations performed through a laparotomy of the lower abdomen are a favorable indication for this procedure. However, these operations require a bilateral blockade. Rather high volumes (15-20 mL each) are needed to achieve adequate distribution of the anesthetic. In particular, when administering a bilateral blockade, the concentration of the local anesthetic must be adjusted to avoid overdosage (33).

## Epidural analgesia

Regional anesthesia procedures used in the vicinity of bone marrow, especially epidural analgesia, are regarded as the gold standard in postoperative analgesia for several abdominal operations. However, the risk-benefit ratio beyond the obstetric setting is a critically debated issue. Therefore, indications for epidural analgesia as a means of perioperative pain management are on the wane (34).

This is because the advantages of epidural anesthesia, as compared to systemic analgesia procedures in combination with other less invasive regional anesthesia procedures (see above), was not very pronounced in recent studies, as in older ones. The reason for this development could be the more consistent use of “ERAS” concepts. Poor bowel preparation and the limitation of preoperative fasting, early enteral nutrition, the omission of drains, and consistent early mobilization of the patient appear to be more significant for the outcome of surgery than the elimination of sympathetic innervation, the opioid-saving effect, and improved analgesia through epidural anesthesia.

On the other hand, according to recent investigations, the risk of relevant undesirable effects of epidural analgesia outside the field of obstetrics, are higher than previously estimated. In an assessment of more than 1.3 million procedures performed in the USA, the risk of spinal hematoma after epidural analgesia procedures for abdominal surgery was reported to be 1:7500 (95% confidence interval: 1:5,663-1:9,736) (35). One of the major unmodifiable risk factors is the Charlson Comorbidity Index. In view of the potentially fatal consequences of epidural hematoma, a careful risk-benefit analysis should be performed, especially in multimorbid patients.

Currently, epidural procedures are no longer recommended as a means of perioperative pain therapy for laparoscopic operations. In multivisceral resection for ovarian cancer, depending on the individual risk profile of the patient, epidural analgesia procedures still constitute a standard approach. However, even for these operations, the positive impact of epidural analgesia on the severity of postoperative pain appears to be limited to the first three days post-surgery. Epidural analgesia was found to have no effect on morbidity, especially the duration of gastrointestinal atony or the incidence of other opioid-induced side effects (36).

The risk-benefit ratio of epidural analgesia procedures is still rated positively in obstetrics. On the one hand, the superiority of peripartum analgesia compared to systemic analgesia procedures is still undisputed. Due to the widespread absence of comorbidities and the highly regulated coagulation system, the risk of severe complications is lower than that in a general postoperative setting; for example, the risk of epidural hematomas is reported to be about 1:150,000 (35). Nevertheless, the suitability of epidural analgesia for postoperative pain management after caesarean section is limited because the injection is given at the level of the lumbar spine, and motor disabilities in the lower extremities cannot be ruled out.

## Preemptive analgesia

Preemptive analgesia is any treatment given to the patient prior to surgery, in order to reduce or prevent subsequent pain. This specifically means that by initiating analgesia (and anaesthesia) prior to the initiation of noxious stimuli, peripheral, and central nervous system pain receptor activation is blocked. This leads to a reduced activity of pain neurotransmitters, processing can be modified, which results in improved short-term and long-term pain control and reduced side effects from narcotic analgesics (37).

Long et al. (38) analyzed a total of 324 studies concerning preemptive analgesia in minimally invasive gynecologic surgery (MIGS). Preemptive blocks, like paracervical, triple antibiotic paste, or pudendal block appear to have the most consistently beneficial effect on postoperative pain in MIGS with an excellent cost-benefit ratio. Preemptive anticonvulsants, ketamine and dexmedetomidine have a positive effect on postoperative pain and opioid use but are limited by side effects. Preemptive dexamethasone, acetaminophen, and NSAIDs have a modest effect on postoperative pain control (38).

Another study group from the US analyzed the effectiveness of preemptive analgesia for pain control in women undergoing total abdominal hysterectomy (39). Sixty-nine randomized controlled trials were included. Concerning nonnarcotic medications, paracetamol, gabapentin, and rofecoxib combined with gabapentin led to improvements in pain assessment compared with placebo and other nonnarcotic medications. The use of preemptive paracetamol, gabapentin, bupivacaine, and phenothiazine resulted in less narcotic usage than placebo (39).

Fast-track surgery (FTS) programs - also known as ERAS - have the aim of ERAS, allowing earlier discharge with improved patient outcomes. Such programs derive their success from their multidisciplinarity, including surgeons, nurses, anesthetists, pain specialists, ward nursing staff, social workers, occupational and physical therapy staff (40). Concerning the anesthesiologic side, the following subjects should be considered: preoperative sedative premedication should be avoided in order to allow early patient mobilization, initiation of early oral feeding and catheter removal (41).

The method of action concerning preemptive analgesia was described earlier in the last section. For preemptive local anaesthesia, a TAP block can be performed just after intubation and just before surgery is commenced. For preemptive analgesia, the administration of Gabapentin can be used. A large number of randomized controlled trials have confirmed decreased analgesic requirements after preoperative gabapentin (42,43).

Further preemptive analgesia is initiated with COX-2 inhibitors given intravenously pre- and intra-operatively (parecoxib) with intravenous paracetamol (44). In addition, intravenous (and oral) paracetamol has opioid sparing effects (45).

Early mobilization and early oral feeding are both central components for a successful FTS.

Both can be better managed through good postoperative pain management. The mobilization is associated with an increased blood circulation and helps to reduce the risk of venous thrombo-embolism. Furthermore, improved pulmonary function with an expansion of the lung bases and better tissue oxygenation should be noted (37).

## Special features of postoperative pain management during puerperium and lactation

Postoperative pain management in the postpartum period is challenging because the patient has a significant need for analgesia, especially after a caesarean section, and the analgesic agents are associated with a risk of toxic effects on the colostrum and breast milk, with potentially harmful effects on the breast-fed infant. At least in German-speaking countries, the need for analgesia in this period is underestimated while the risks for the newborn are overestimated. This miscalculation does not restrict the use of analgesic treatment on the part of the treating physicians alone. Patients also tolerate a high level of pain before they ask for analgesics that, in their estimation, may potentially affect breast milk. This is because of their (unwarranted) concern regarding toxic effects on the newborn. Sufficient analgesia is especially important during the puerperium, because it is a prerequisite for early mobilization of the patient and the avoidance of postpartum thrombosis, and is also a predictor of the success of breastfeeding. Last but not least, one must consider the fact that the risk and severity of puerperal depression are linked with the severity of postpartum pain. These ideas mentioned before, form a contrast to pain management in oncological gynecology. In many cases, oncological patients have become accustomed to their pain over a long period of time and often take analgesics as permanent medication - sometimes in increasing doses due to increasing pain or an incipient habituation effect. This is in contrast to obstetric patients who experience a new form of pain from complete health.

## Blood-milk barrier

**Figure 4:**
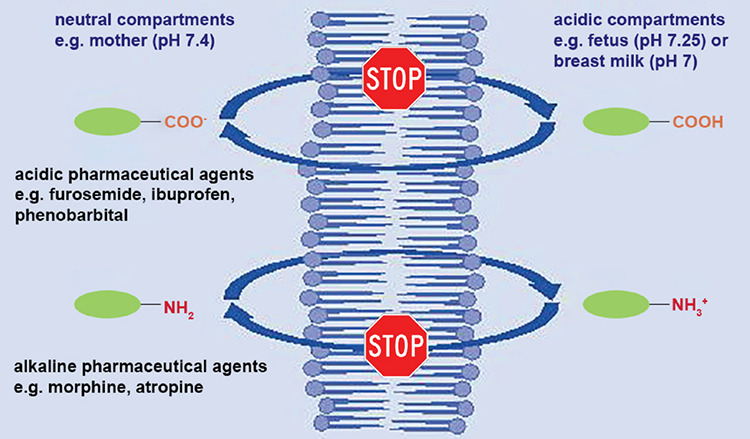
Ion trap. Acids absorb a hydrogen ion in an acidic environment and are not charged with it (= lipophilic = can pass through membranes). In a neutral or alkaline environment, they release a hydrogen ion and are charged with it (= hydrophilic = cannot pass through membranes). Therefore, they accumulate in an alkaline or neutral environment. Alkaline pharmaceutical agents behave in the opposite manner and accumulate in acidic compartments such as the fetus or breast milk [extracted from (48)]

The blood-milk barrier has similar properties as the placental barrier and hinders the entry of pharmaceutical agents into breast milk to a limited extent. In many cases, small molecules and lipophilic substances can be freely transferred to breast milk. The pH-dependent load of the substances is of crucial importance (Figure 4). Acidic substances, such as ibuprofen, are virtually absent from breast milk, whereas alkaline substances, such as opioids, accumulate in breast milk. In addition to the milk/plasma ratio, which describes the extent of transfer of a pharmaceutical agent into breast milk, the quantity of breast milk ingested by the newborn infant is also a significant determinant of the neonatal dose. While infants ingest breast milk at a rate of about 150 mL/kg body weight, the quantity of colostrum is much less during the first few days postpartum. Consequently, even if the mother is given high doses of opioids during the puerperium, the load on the newborn infant is negligible. For instance, even after mothers were given high doses of oxycodone during the first 48 hours postpartum, only one of 41 breast-fed infants had detectable and clinically relevant oxycodone levels in plasma (46).

Regrettably, we lack robust data for many pharmaceutical agents and their use in the postpartum period and during lactation. The largest body of clinical experience is available for paracetamol (M/P coefficient 1), ibuprofen (M/P coefficient 0.008) and fentanyl, which is widely used in English-speaking countries. However, due to similar pharmacodynamics and pharmacokinetics, this experience is also applicable to other opioids such as tramadol, piritramide or oxycodone (M/P coefficient 2-3.6). Codeine and pethidine are exceptions. Their use should be avoided during pregnancy and lactation because of their cumulative metabolic effects. The establishment of a maximal dose of opioids during the postpartum period is not meaningful because neither maternal plasma levels nor levels of the substances in breast milk are correlated with the doses of opioids (46).

## Procedure-specific pain management in obstetrics and gynecology

The surgical spectrum of obstetrics and gynecology is wide. No general concept of postoperative pain management can be recommended for these specialties. Rather, it would be meaningful to develop procedure-specific concepts of postoperative pain management in cooperation with the involved clinical departments (obstetrics and gynecology, anesthesia, nursing staff, and pediatrics if applicable). We have evidence-based recommendations of the European Society of Regional Anaesthesia and Pain Therapy for some operations. However, some of these are outdated. In view of the above mentioned therapy options, one alternative for procedure-specific pain management in obstetrics and gynecology is listed in Table 1. When aligning these therapy concepts to specific clinical conditions, one must consider the fact that the clinician’s experience of the various procedures and pharmaceutical agents could be more important than the theoretical advantages reported in published studies.

## Conclusion

The current non-optimum quality of postoperative pain therapy is not restricted to German-speaking countries alone. Furthermore, obstetrics and gynecology are not excepted from this statement. Rather, these surgical specialties are associated with the most severe postoperative pain.

Current concepts of postoperative pain management are largely based on the administration of systemic non-opioid and opioid analgesics. These are supplemented with regional anesthesia procedures, and in some cases with other pharmaceutical analgesics. Based on the existing body of evidence, it would be appropriate to develop procedure-specific analgesia concepts that take the special characteristics of the specialties into account. In general, however, the clinician’s personal experience of the procedures and substances could be more important than the theoretical advantages reported in published studies.

## Figures and Tables

**Table 1:**
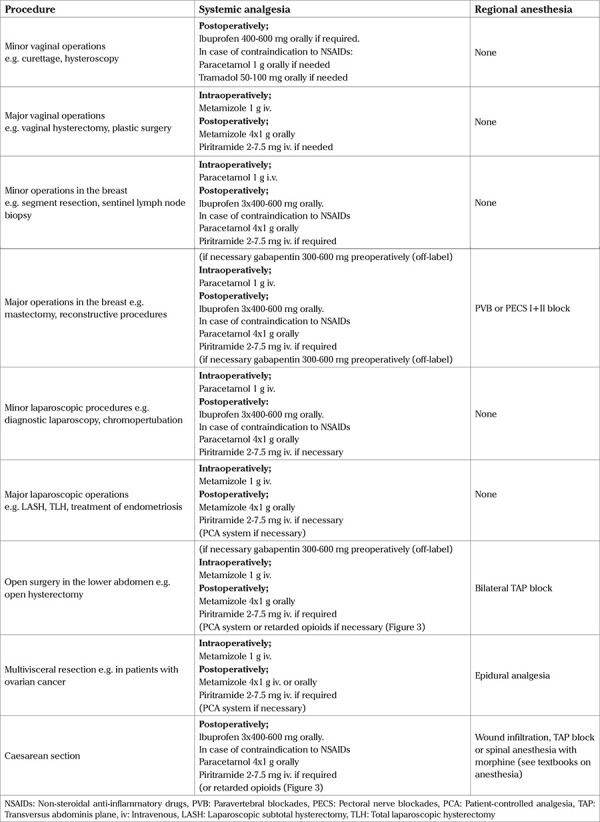
Concept of procedure-specific postoperative pain management in obstetrics and gynecology. The use of treatment concepts must be aligned to the individual patient’s risk profile
